# Occupational hypersensitivity pneumonitis in a koji brewer

**DOI:** 10.1002/ccr3.1317

**Published:** 2018-01-19

**Authors:** Takashi Ishiguro, Shoko Kawai, Ayako Kojima, Yoshihiko Shimizu, Katsuhiko Kamei, Noboru Takayanagi

**Affiliations:** ^1^ Department of Respiratory Medicine Saitama Cardiovascular and Respiratory Center Saitama Japan; ^2^ Department of Pathology Saitama Cardiovascular and Respiratory Center Saitama Japan; ^3^ Faculty of Mycology Chiba University Chiba Japan

**Keywords:** *Aspergillus flavus*, *Aspergillus oryzae*, hypersensitivity pneumonitis, koji, *β*‐D‐glucan

## Abstract

Koji is a fermenting agent used in many traditional Japanese foods*,* and *Aspergillus oryzae* is the most frequently used microorganism in koji production. Few cases of hypersensitivity pneumonitis due to *A. oryzae* have been reported. However, physicians should recognize the disease because of the increasing globalization of food production.

## Introduction

Hypersensitivity pneumonitis (HP) is an allergic, diffuse interstitial lung disease caused by repeated inhalation of extrinsic materials resulting in granuloma formation and alveolitis. The main types seen in Japan are summer‐type HP due to *Trichosporon asahii* and bird‐related HP, but occupational types of HP such as farmer's lung, isocyanate‐related HP, and mushroom worker's HP are also known 1.

We recently experienced a patient with occupational HP caused by exposure to *Aspergillus oryzae* in a koji brewery. Koji is needed to make soy sauce (*shoyu*), miso, and a range of other traditional Japanese foods. Koji is also brewed and used in other countries, reflecting the recent globalization of food production, and thus, recognition of HP related to koji exposure is important.

## Case Report

A 63‐year‐old woman working in a koji brewery presented to our hospital with a prolonged cough for 20 years but that had been increasing since 2015. In March 2016, she developed dyspnea on effort, and she presented to our hospital in June 2016. She had no past histories including allergic disorders and did not smoke or drink alcohol. She had not experienced episodic wheezes or rhonchi. She had been working as a koji brewer in a factory of her family's koji‐brewing business for 30 years.

**Figure 1:**
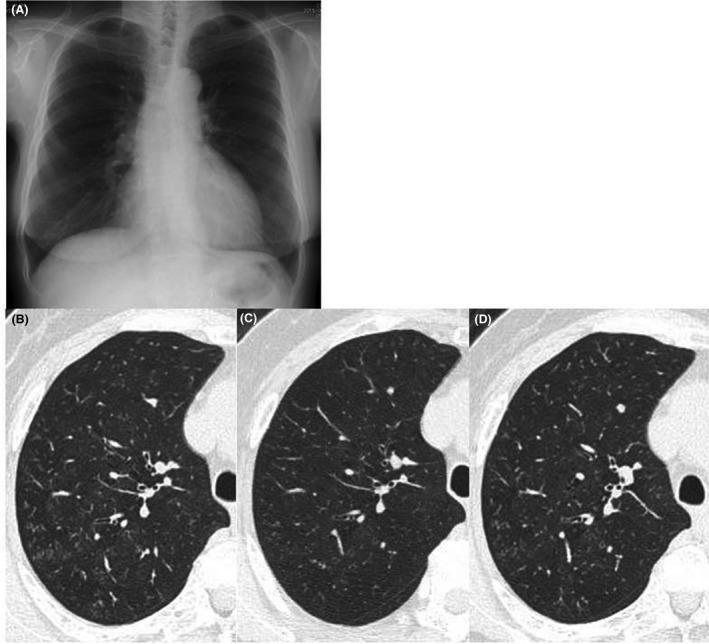
Chest imaging. Chest X‐ray on initial presentation showed no abnormal findings (A), but chest computed tomography showed centrilobular nodules and ground‐glass opacities (B). After the patient stopped working at the koji brewery, the abnormal shadows improved on computed tomography (C); however, they relapsed after she returned to the brewery (D).

On presentation, mild fine crackles were heard, and her respiratory rate was 17 per minute. Body temperature was 36.2°C. Results of pulmonary function tests including vital capacity, forced expiratory volume in 1 sec (FEV_1_), and FEV_1_/forced vital capacity were within normal range. The FEV_1_ did not change by bronchodilator inhalation. Her diffusion capacity of carbon monoxide was not measured. Blood gas analysis under ambient air showed a pH of 7.42, PaCO_2_ of 39.5 Torr, PaO_2_ of 79.4 Torr, and HCO_3_‐ of 25.2 mmol/L. Laboratory test results were as follows: white blood cell count (WBC), 4000/mm^3^; hemoglobin, 12.5 g/dL; platelets, 22.4 × 10^4^/mm^3^; serum total protein, 7.5 g/dL; albumin, 4.2 g/dL; creatinine, 0.6 mg/dL; Na, 141 mmol/L; Cl, 104 mmol/L; K, 4.2 mmol/L; lactate dehydrogenase, 234 IU/L; C‐reactive protein (CRP), 0.1 mg/dL; KL‐6, 316 U/mL; IgE, 385 IU/mL; *β*‐D‐glucan, 30.5 pg/mL (normal range <11 pg/mL); and *Aspergillus* antigen (galactomannan antigen), 6.4. Serum anti‐*Trichosporon asahii* antibody, IgA antibodies against *Mycobacterium avium*, and interferon‐gamma releasing assay were all negative. Chest X‐ray (Fig. 1A) showed no abnormal shadows, but computed tomography (Fig. 1B) showed ground‐glass opacities and centrilobular nodules in both upper lobes.

Bronchoalveolar lavage could not recover a sufficient amount of saline, and transbronchial lung biopsy showed alveolitis without granuloma formation. *A. oryzae* was cultured from the bronchoalveolar lavage fluid, and serum precipitating antibody against *A. oryzae* was positive. *A. oryzae* was repeatedly isolated from her factory. After admission, her symptoms improved, and her PaO_2_ under ambient air improved to 97.5 Torr, although pulmonary function tests remained unchanged. The ground‐glass opacities and centrilobular nodules on computed tomography (CT) improved (Fig. 1C), and her serum *β*‐D‐glucan value decreased to <11 pg/mL. Thereafter, she returned to her factory, after which her cough relapsed, her PaO_2_ decreased to 79.7 Torr, and she redeveloped centrilobular nodules on chest CT (Fig. 1D). Although her WBC counts and serum CRP values did not worsen, we diagnosed her as having HP due to *A. oryzae*. Her serum *β*‐D‐glucan value was elevated to 28.8 pg/mL. She has stopped working at the factory, but her family has continued to brew koji. She continues to be followed on an outpatient basis, and her HP has not relapsed.

## Discussion

The present patient developed HP due to *A. oryzae* at a koji‐brewing factory. Her HP improved by stopping her work, but it relapsed upon her return to working in the factory. Differential diagnosis of her prolonged cough included asthma, but the absence of abnormal pulmonary function, absence of the response of FEV_1_ to bronchodilator inhalation, and absence of episodic wheezes or rhonchi, and the existence of ground‐glass opacities in lung fields, which improved by hospitalization and worsened by exposure to the factory, and alveolitis indicated asthma to be unlikely.

Koji is made by sprinkling *tane‐koji*, literally “seed koji,” over steamed rice, barley, or soybeans and cultivating the fungus under temperature conditions suitable for its growth. As the fungus propagates, enzymes break down the grains’ starch and proteins into sugars and amino acids. Because of these characteristics, koji is used to produce such items as fermented foods, soy sauce, miso, sweet rice wine, vinegar, and *shochu* (a distilled alcoholic beverage), and koji is embedded in the food customs of Japan. Representative koji‐producing species include *A. oryzae*,* A. tamarii*,* A. awamori*, and *A. glaucus*. Producing and brewing koji require special skills that have been handed down through the generations in family businesses. Fortunately, our patient's family could continue production after our patient gave up her job, but it could have potentially been a large problem if other family members could not take over the work because the loss of koji‐brewing skills has become a problem in Japan as the population with this skill set decreases.

The treatment of HP is to avoid inhalation of the fungi, but this is difficult for koji brewers because they need to handle the fungi directly. Our patient's symptoms and laboratory and radiological findings, including those of high‐resolution CT, improved by leaving the factory. When patients cannot leave the place where they developed HP, management of the condition may be troublesome. Patients need preventive measures to avoid future contact with the offending antigen, such as improved air conditioning at the worksite, wearing of masks, wetting the compost to keep it from flying about, and supplying pretreated compost that requires no personal preparation. The usefulness of dust masks, for example, has been regarded as controversial with effective 2 and ineffective 3 results.

*Aspergillus oryzae* is a member of the *A. flavus* group. To our knowledge, only a limited number of cases of HP due to *A. flavus* and *A. oryzae* have been reported 4, 5, 6, 7, and other cases of allergic bronchopulmonary aspergillosis or occupational asthma have also been reported 8, 9, 10. The number of reports of allergic diseases caused by *A. oryzae* seems to be limited when compared to that caused by *A. fumigatus*. The strength or amount of antigenicity may affect this 11, but detailed reasons are unclear. *A. oryzae* was isolated from bronchoalveolar lavage fluid in the present patient, which is uncommon in HP patients. Our patient worked several hours in the brewery before undergoing bronchoscopy, and that might be the cause. In addition, a large amount of inhaled *A. oryzae* would be expected in the context of her job.

The serum *β*‐D‐glucan value and *Aspergillus* antigen index were elevated on presentation of our patient, and these were altered by our patient's avoiding and then returning to the factory. However, she did not have a fever or an increased peripheral WBC count or serum CRP value when her *β*‐D‐glucan value and *Aspergillus* antigen index increased, and no findings suggesting invasive infection were found. Although one case of HP with an increased *β*‐D‐glucan value in bronchoalveolar lavage fluid was reported 12, no cases with increased serum values of *β*‐D‐glucan or *Aspergillus* antigen index have been reported. *β*‐D‐glucan is an attractive antigen in that it is found in a broad range of fungal agents, including the commonly encountered agents *Candida* spp., *Aspergillus* spp., and *Pneumocystis jirovecii*. Cross‐reactions with certain hemodialysis filters, beta‐lactam antimicrobials, and immunoglobulins, which raise concerns about false‐positive tests, have also been described but our patient had not been exposed to these. There have been reports showing *β*‐glucan absorption through the intestine 13, 14, 15 but no reports of its absorption through bronchial wall mucosa. Thus, we speculate that a large amount of mucosal fungi was inhaled by our patient, and some of these fungi were swallowed into the digestive system and then absorbed from the intestine, resulting in her elevated blood *β*‐D‐glucan value. Further studies are needed in which the serum values of *β*‐D‐glucan or the *Aspergillus* antigen index are measured in koji brewery workers.

We report a patient with occupational HP due to *A. oryzae* exposure. Physicians should recognize the disease because the globalization of food products has spread the production of koji around the world.

## Authorship

TI: is the guarantor of the manuscript, taking responsibility for the integrity of the work as a whole, from inception to published article. SK, AK, YS, KK, and NT: aggregated the data, created the figures, and helped draft the discussion of the manuscript.

## Conflict of Interest

None declared.
